# Safety, Tolerability, and Pharmacokinetics of MEDI8897, the Respiratory Syncytial Virus Prefusion F-Targeting Monoclonal Antibody with an Extended Half-Life, in Healthy Adults

**DOI:** 10.1128/AAC.01714-16

**Published:** 2017-02-23

**Authors:** M. Pamela Griffin, Anis A. Khan, Mark T. Esser, Kathryn Jensen, Therese Takas, Martin K. Kankam, Tonya Villafana, Filip Dubovsky

**Affiliations:** a MedImmune (AstraZeneca), Gaithersburg, Maryland, USA; b Vince and Associates Clinical Research, Overland Park, Kansas, USA

**Keywords:** RSV, MEDI8897, pharmacokinetics, healthy adults, infants, lower respiratory tract infections, respiratory syncytial virus

## Abstract

Prevention of respiratory syncytial virus (RSV) illness in infants is a major public health priority, but there is no approved vaccine. Palivizumab is a monoclonal antibody that provides RSV prophylaxis but requires 5 monthly injections and is approved only for infants who experience the greatest morbidity and mortality from RSV. Thus, there remains a significant unmet medical need for prevention of RSV disease in healthy infants. MEDI8897 is a recombinant human RSV monoclonal antibody with a modified Fc region that extends its half-life and is being developed as RSV prophylaxis for all infants. In this phase 1, first-in-human, placebo-controlled study, 136 healthy adults were randomized to receive a single dose of MEDI8897 (*n* = 102) or placebo (*n* = 34) in 1 of 5 cohorts (300, 1,000, or 3,000 mg intravenously or 100 or 300 mg intramuscularly [i.m.]) and were monitored for 360 days. The mean half-life of MEDI8897 was 85 to 117 days across dose groups, and bioavailability after 300-mg i.m. dose administration was 77%. Time to maximum concentration following i.m. dosing was 5 to 9 days. Antidrug antibody (ADA) responses were detected in a similar proportion of placebo (15.2%) and MEDI8897 (13.7%) recipients. The safety profile of MEDI8897 was similar to that of the placebo. These results support clinical studies of the i.m. administration of a single dose of MEDI8897 in the target population of infants to provide protection for the duration of the RSV season. (This study has been registered at ClinicalTrials.gov under identifier NCT02114268.)

## INTRODUCTION

Respiratory syncytial virus (RSV) causes annual worldwide epidemics and is the most common cause of lower respiratory tract disease among infants and young children (1–7).

Ninety percent of children are infected with RSV in the first 2 years of life, and up to 40% of them will develop lower respiratory tract infection (LRTI) with the initial episode (8–10). In 2005, an estimated 33.8 million (95% confidence interval [CI], 19.3 to 46.2 million) new episodes of RSV-associated LRTI occurred worldwide in children younger than 5 years of age, resulting in an estimated 66,000 to 199,000 deaths, most of which occurred in the developing world (5).

Despite almost 50 years of attempted vaccine development (11) and extensive ongoing work (clinicaltrials.gov), there is still no licensed safe and effective vaccine. While RSV prevention exists in the form of a specific RSV immunoglobulin G (IgG) monoclonal antibody (palivizumab [Synagis]) requiring 5 monthly injections, it is licensed only for infants who are at risk for experiencing the greatest morbidity and mortality from RSV (preterm infants of ≤35 weeks gestational age [wGA], children with chronic lung disease of prematurity, and children with hemodynamically significant congenital heart disease). In addition, due to the cost of palivizumab prophylaxis, the American Academy of Pediatrics has suggested further restrictions, and palivizumab is not recommended for healthy preterm infants who are ≥29 wGA (12). Currently, there is no approved RSV prophylaxis for the broader population of healthy newborns and infants, and there is no effective treatment for active RSV infection. The current standard of care for infants and children who develop serious RSV illness is supportive care.

MEDI8897, a recombinant human IgG1 kappa monoclonal antibody that targets the prefusion conformation of the RSV F protein (13), is under clinical development for the prevention of RSV-associated LRTI in all infants. MEDI8897 is derived from D25, a human monoclonal antibody that exhibits approximately 100-fold greater potency than palivizumab *in vitro* (14), and is engineered with a triple-amino-acid (M252Y/S254T/T256E [YTE]) substitution within its Fc region. The YTE substitution enhances the binding of IgG1 to the neonatal Fc receptor (FcRn) under the acidic conditions (pH 6.0) of the lysosome. This prevents degradation and increases recirculation to the surface of the cell, thereby prolonging the serum half-life of the antibody (15, 16). Previously, it was shown that palivizumab clearance increased slightly from 10.2 ml/day to 11.9 ml/day as a function of postmenstrual age (wGA plus chronological age), ranging from 7 to 18 months, and the half-life ranged from 17 to 26.8 days (17). As the half-life of palivizumab is approximately 20 days in children less than 24 months of age (18), monthly administration as an intramuscular (i.m.) injection throughout the RSV season is required, and therefore its use in a broader healthy infant population is not feasible. MEDI8897 provides an opportunity to protect all infants from RSV disease based on an increased potency and extended half-life that supports once-per-RSV-season dosing. This first-in-human study in healthy adult volunteers was designed to evaluate the pharmacokinetics (PK) and safety profile of MEDI8897 before initiating a clinical study in infants.

## RESULTS

### Subject disposition.

**FIG 1:**
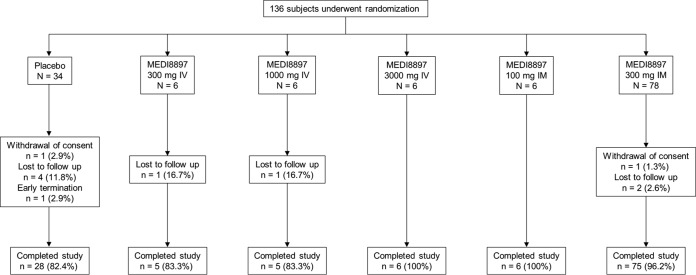
Subject disposition.

Of the 136 subjects randomized to receive placebo or MEDI8897, 125 (91.9%) completed the study (Fig. 1). Among the 11 subjects discontinuing the study, 6 were randomized to the placebo group, while 5 were randomized to receive MEDI8897. Of these subjects, 8 were lost to follow-up, 2 withdrew consent, and 1 was withdrawn from the study due to noncompliance.

Overall, the demographics and baseline characteristics of subjects in the placebo and MEDI8897 groups were similar (Table 1). Female subjects constituted 52.9% of the MEDI8897 group and 55.9% of the placebo group. Just over half of the subjects were African American (54.9% in the MEDI8897 group and 61.8% in the placebo group).

**TABLE 1:** Demographics and baseline characteristics

Characteristic^*a*^	Value(s) at MEDI8897 dose of:						
	300 mg i.v. (*n* = 6)	1,000 mg i.v. (*n* = 6)	3,000 mg i.v. (*n* = 6)	100 mg i.m. (*n* = 6)	300 mg i.m. (*n* = 78)	Total (*n* = 102)	Placebo (*n* = 34)
Mean age, yr (SD)	34.5 (8.6)	34.3 (5.6)	33.5 (6.7)	30.7 (7.8)	30.3 (7.9)	31.0 (7.8)	29.2 (8.6)
Female, *n* (%)	4 (66.7)	2 (33.3)	3 (50.0)	6 (100)	39 (50.0)	54 (52.9)	19 (55.9)
Race, *n* (%)							
African American	4 (66.7)	2 (33.3)	4 (66.7)	2 (33.3)	44 (56.4)	56 (54.9)	21 (61.8)
White	2 (33.3)	4 (66.7)	2 (33.3)	4 (66.7)	34 (43.6)	46 (45.1)	12 (35.3)
Native Hawaiian or other Pacific Islander	0	0	0	0	0	0	1 (2.9)
Mean wt, kg (SD)	85.2 (20.1)	75.3 (18.5)	82.2 (16.4)	71.9 (13.2)	77.8 (14.3)	78.0 (14.9)	80.5 (16.9)
Mean BMI, kg/m^2^ (SD)	28.5 (6.0)	25.4 (5.2)	29.6 (6.4)	30.9 (9.6)	26.9 (5.6)	27.3 (6.0)	27.5 (4.6)

a BMI, body mass index.

### Pharmacokinetics.

**FIG 2:**
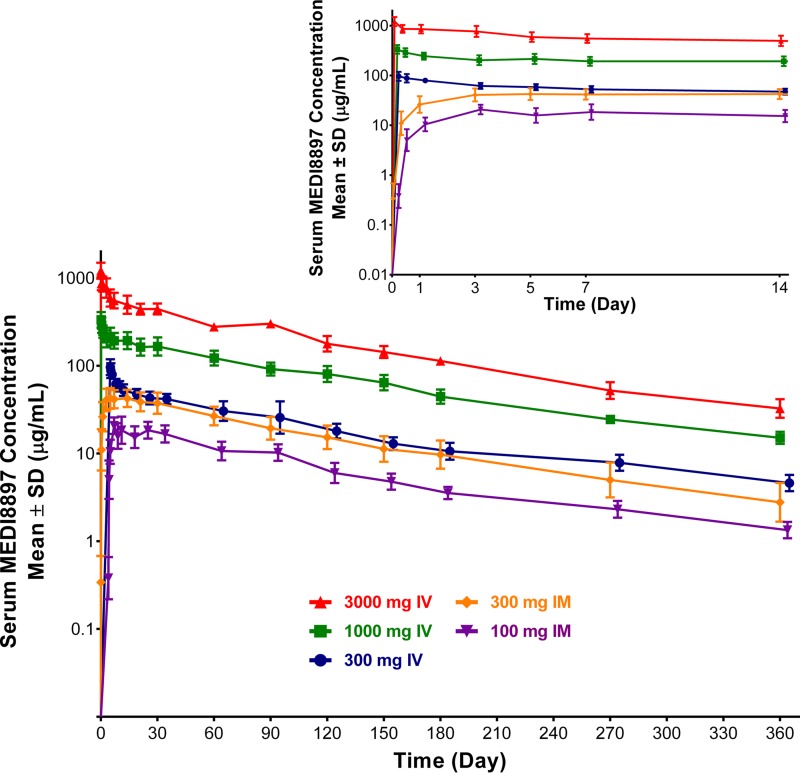
Mean serum MEDI8897 concentrations after a single i.v. or i.m. dose. Data have been jittered. Error bars represent the standard deviations.

Following intravenous (i.v.) administration of MEDI8897 at 300 to 3,000 mg, mean serum PK parameters, including peak serum concentration (*C*_max_) and area under the concentration-time curve from time zero extrapolated to infinity (AUC_0–∞_), increased in an approximately dose-proportional manner (Table 2 and Fig. 2). In subjects receiving an i.v. dose, mean clearance (CL) was 40.3 to 47.6 ml/day, while mean terminal half-life (*t*_1/2_) was 90 to 117 days.

**TABLE 2:** MEDI8897 mean pharmacokinetic parameter values^*a*^

Parameter	Mean value (SD) at MEDI8897 dose of^*b*^:				
	300 mg i.v. (*n* = 6)	1,000 mg i.v. (*n* = 6)	3,000 mg i.v. (*n* = 6)	100 mg i.m. (*n* = 6)	300 mg i.m. (*n* = 78)
*t*_max_, days	0.0780 (0.134)	0.0590 (0)	0.209 (0.131)	5.46 (3.90)	9.42 (7.39)
*C*_max_, μg/ml	97.0 (21.2)	334 (74.8)	1,160 (277)	20.4 (6.00)	47.5 (12.5)
AUC_0-∞_, μg · day/ml	6,710 (1,450)	25,300 (4,301)	63,600 (6,620)	2,250 (402)	5,190 (1,670)
*t*_1/2_, days	117 (22.8)	92.0 (11.6)	89.8 (16.3)	103 (11.6)	85.3 (26.3)
CL,^*c*^ ml/day	46.1 (7.96)	40.3 (6.20)	47.6 (5.04)	45.5 (7.02)	64.6 (24.4)
*V_z_*,^*d*^ liters	7.69 (1.91)	5.43 (1.45)	6.14 (1.13)	6.80 (1.67)	7.46 (2.54)
*V_ss_*,^*e*^ liters	6.60 (1.20)	5.16 (1.27)	5.58 (1.03)	5.84 (1.45)	6.90 (1.74)

a AUC_0-∞_, area under the curve from time zero to infinity; CL, clearance; *C*_max_, peak plasma concentration; i.m., intramuscular; i.v., intravenous; SD, standard deviation; *t*_1/2_, terminal half-life; *t*_max_, time to peak plasma concentration; *V_z_*, volume of distribution.

b *n* = 5 for calculation of AUC_0-∞_, *t*_1/2_, CL, and *V_z_* for all i.v. and 100-mg i.m. cohorts. *n* = 75 for calculation of AUC_0-∞,_ *t*_1/2_, CL, and *V_z_* for the 300-mg i.m. cohort.

c CL/*F* (extravascular) for the i.m. dose groups, where *F* is bioavailability following i.m. administration.

d *V_z_*/*F* (extravascular) for the i.m. dose groups.

e *V_ss_*/*F* (extravascular) for the i.m. dose groups.

Among those receiving an i.m. dose of MEDI8897 at 100 or 300 mg, mean *C*_max_ and AUC_0–∞_ demonstrated a slightly less than dose-proportional increase (Table 2 and Fig. 2). Mean CL ranged from 45.5 to 64.6 ml/day, and mean *t*_1/2_ was 85.3 to 103 days among those receiving an i.m. dose. Due to an observation of relatively higher CL in a small subset of individuals at the 300-mg-i.m.-dose cohort, the mean CL was higher than the estimated CL in other dosing cohorts and resulted in a less than proportional increase in the AUC_0–∞_. Systemic bioavailability of MEDI8897 was estimated to be 77% following the 300-mg i.m. dosing.

### RSV neutralizing activity.

**FIG 3:**
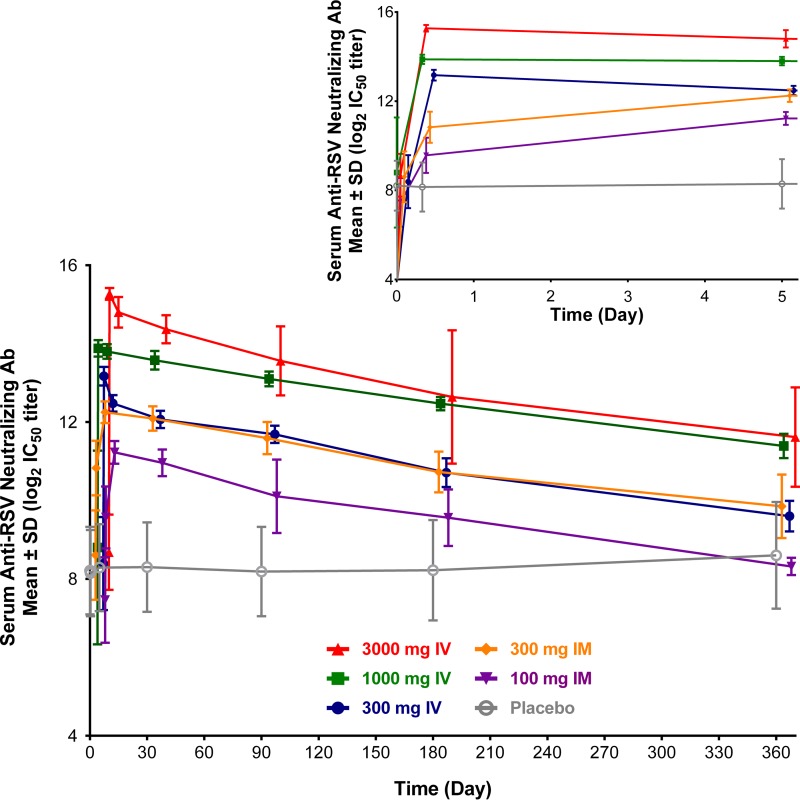
Anti-RSV neutralizing antibody titers after a single i.v. or i.m. dose of MEDI8897 or placebo. Data points represent the mean anti-RSV A2 neutralizing antibody titers on a log_2_ scale. Data have been jittered. Error bars represent the standard deviations. Ab, antibody.

To compare the levels of endogenous anti-RSV neutralizing antibodies before administration of MEDI8897 and those afforded by MEDI8897, anti-RSV neutralizing antibodies were measured at baseline and through day 361. Serum anti-RSV neutralizing antibodies were detected prior to dose administration in all subjects receiving MEDI8897 or placebo (Fig. 3). At baseline, RSV neutralizing antibody titers were similar across all dosing groups. The mean log_2_ titers were 8.2, 8.4, 8.8, 8.7, 7.5, and 8.6 for the groups receiving placebo or MEDI8897 at 300 mg i.v., 1,000 mg i.v., 3,000 mg i.v., 100 mg i.m., and 300 mg i.m., respectively. In individual subjects, baseline RSV neutralization titers ranged from 5.1 to 11.7 log_2_.

Except for 1 subject who had a 4-fold increase in neutralizing antibody titer, possibly due to an RSV infection or exposure, subjects in the placebo group had stable titers for the duration of the study (mean titer range, 8.2 to 8.6 log_2_). Among those receiving MEDI8897, RSV neutralizing antibody titers increased in a dose-dependent manner (Fig. 3). The highest titers were detected 8 h postinfusion in those receiving an i.v. dose and on day 6 in those receiving an i.m. dose. At 8 h postinfusion, the geometric mean fold rise in titers was 0.96, 27.4, 33.8, and 96.0 among subjects receiving placebo or MEDI8897 at 300 mg i.v., 1,000 mg i.v., and 3,000 mg i.v., respectively. The geometric mean fold rise in titers on day 6 was 1.1, 13.6, and 12.5 for placebo, MEDI8897 at 100 mg i.m., and MEDI8897 at 300 mg i.m., respectively. On day 31, neutralizing antibody titers had increased ≥4-fold relative to baseline levels in 100% of subjects receiving an i.v. dose of MEDI8897 and in 83% and 91% of subjects receiving 100 mg i.m. and 300 mg i.m. MEDI8897, respectively. This ≥4-fold increase in neutralizing antibody titers from baseline persisted until day 181 in 60%, 80%, 83%, 50%, and 55% of subjects receiving 300 mg i.v., 1,000 mg i.v., 3,000 mg i.v., 100 mg i.m., and 300 mg i.m. MEDI8897, respectively. Neutralizing antibody titers were lower in the 300-mg i.m. group than in the 300-mg i.v. group at 8 h following administration but were comparable at day 6 through the end of the study. In general, administration of MEDI8897 resulted in increased levels of neutralizing antibodies that were maintained above background levels for up to 1 year.

### ADA response.

Antidrug antibody (ADA) was detected at baseline in 5 of 102 (4.9%) MEDI8897 recipients and in 3 of 34 (8.8%) placebo recipients. Postbaseline ADA was detected in 14 of 102 subjects (13.7%) receiving MEDI8897 and in 5 of 33 subjects (15.2%) receiving placebo, with a maximum titer of 1:800 and 1:400, respectively.

### Safety.

Treatment-emergent adverse effects (AEs) were reported for 64 of 102 (62.7%) subjects receiving MEDI8897 and 21 of 34 subjects (61.8%) receiving placebo (Table 3), the majority of which were mild or moderate in severity. Overall, the most common AEs among those receiving MEDI8897 were upper respiratory tract infection (18.6%), headache (8.8%), urinary tract infection (5.9%), contact dermatitis (4.9%), musculoskeletal pain (4.9%), nausea (4.9%), and vomiting (4.9%) (Table 4). Among those receiving placebo, the most frequent AEs were headache (17.6%), upper respiratory tract infection (8.8%), nausea (5.9%), increased serum creatine phosphokinase level (5.9%), and paresthesia (5.9%). AEs occurred within 2 days of dosing in 21 of 102 MEDI8897 recipients (20.6%) and 10 of 34 placebo recipients (29.4%). AEs that occurred within 2 days of dosing in more than 1 MEDI8897 recipient were headache (*n* = 4), nausea (*n* = 3), abdominal pain (*n* = 2), and constipation (*n* = 2), and those occurring in more than 1 placebo recipient were headache (*n* = 4) and paresthesia (*n* = 2). Two serious AEs (gunshot wound and appendicitis) were reported in 2 subjects receiving 300 mg i.m. MEDI8897, both of which were considered by the investigator to be unrelated to the investigational product. No deaths or adverse effects of special interest (AESIs) occurred during this study. In addition, there were no study discontinuations due to AEs or serious AEs (SAEs).

**TABLE 3:** Safety summary

Event^*a*^	No. (%) of subjects with ≥1 event at MEDI8897 dose of:						
	300 mg i.v. (*n* = 6)	1,000 mg i.v. (*n* = 6)	3,000 mg i.v. (*n* = 6)	100 mg i.m. (*n* = 6)	300 mg i.m. (*n* = 78)	Total (*n* = 102)	Placebo (*n* = 34)
Any TEAE	3 (50.0)	3 (50.0)	5 (83.3)	4 (66.7)	49 (62.8)	64 (62.7)	21 (61.8)
AE related to study drug	0	3 (50.0)	2 (33.3)	2 (33.3)	11 (14.1)	18 (17.6)	10 (29.4)
AE severity grade of ≥3	0	0	0	0	2 (2.6)	2 (2.0)	1 (2.9)
SAE	0	0	0	0	2 (2.6)	2 (2.0)	0
SAE and/or AE severity grade of ≥3	0	0	0	0	3 (3.8)	3 (2.9)	1 (2.9)
NOCD	0	0	0	0	0	0	1 (2.9)
Death	0	0	0	0	0	0	0

a AE, adverse event; NOCD, new onset of chronic disease; SAE, serious adverse event; TEAE, treatment-emergent adverse event.

**TABLE 4:** Treatment-emergent adverse events occurring in >2% of subjects receiving MEDI8897

AE	No. (%) of subjects with AE at MEDI8897 dose of:						
	300 mg i.v. (*n* = 6)	1,000 mg i.v. (*n* = 6)	3,000 mg i.v. (*n* = 6)	100 mg i.m. (*n* = 6)	300 mg i.m. (*n* = 78)	Total (*n* = 102)	Placebo (*n* = 34)
Upper respiratory tract infection	1 (16.7)	1 (16.7)	0	1 (16.7)	16 (20.5)	19 (18.6)	3 (8.8)
Headache	0	1 (16.7)	1 (16.7)	0	7 (9.0)	9 (8.8)	6 (17.6)
Dermatitis (all)	1 (16.7)	0	2 (33.3)	0	4 (5.1)	7 (6.9)	2 (5.9)
Urinary tract infection	0	0	0	2 (33.3)	4 (5.1)	6 (5.9)	1 (2.9)
Musculoskeletal pain	0	0	1 (16.7)	0	4 (5.1)	5 (4.9)	1 (2.9)
Nausea	0	0	2 (33.3)	0	3 (3.8)	5 (4.9)	2 (5.9)
Vomiting	0	0	0	0	5 (6.4)	5 (4.9)	1 (2.9)
Cough	0	0	0	0	4 (5.1)	4 (3.9)	0
Abdominal pain	0	0	0	0	3 (3.8)	3 (2.9)	0
Arthralgia	0	0	0	0	3 (3.8)	3 (2.9)	0

Overall, 18 subjects (17.6%) in the MEDI8897 group and 10 subjects (29.4%) in the placebo group experienced AEs that were considered treatment related. The most frequently reported treatment-related AEs were headaches, occurring in 3 (2.9%) and 4 (11.8%) subjects of the MEDI8897 and placebo groups, respectively. All treatment-related AEs were of mild severity in the MEDI8897 group and of mild or moderate severity in the placebo group.

Adverse events with a grade of severity of ≥3 were reported in 2 subjects receiving MEDI8897 at 300 mg i.m. (gunshot wound and eye injury) and in 1 receiving placebo (blood creatine phosphokinase increased) (Table 3), all of which were considered unrelated to the treatment administered. For the duration of the study, a single new-onset chronic diseases (NOCDs) of type 2 diabetes was reported in 1 subject receiving placebo and was considered unrelated to treatment.

## DISCUSSION

This is the first clinical study with MEDI8897, a fully human anti-RSV prefusion F monoclonal antibody with increased potency and an extended half-life. These modifications provide the potential for a vaccine-like strategy for the prevention of RSV disease in all infants. RSV has been recognized globally by the World Health Organization and others as the leading cause of serious LRTI in infants. Although extensive efforts have been made toward the development of an RSV vaccine in a number of clinical trials, there are none that are currently approved in any country. The only RSV prevention available is with palivizumab, which is licensed for infants and children who are at high risk of serious RSV disease (18). Palivizumab has a mean half-life of approximately 20 days, which is expected for a standard IgG1 monoclonal antibody, and requires 5 monthly doses through the typical RSV season (17) to achieve and maintain a protective level of neutralizing antibody. With these dosing requirements, application to a broader population of healthy infants is not feasible. MEDI8897 has advantages over palivizumab of increased potency and a long half-life, providing the possibility for protection from RSV disease with a single i.m. dose.

In this study, we have shown that, with the YTE substitution, MEDI8897 has an extended mean half-life of 85 to 117 days in healthy adults. The YTE modification, however, did not affect other PK parameters, such as time to maximum concentration or bioavailability, as the observed values were within the ranges expected for a typical IgG1 monoclonal antibody. Furthermore, administration of MEDI8897 results in high levels of RSV neutralizing antibodies that are maintained for more than 150 days in many subjects. A number of key observations in infants have demonstrated that high levels of RSV neutralizing antibodies either maternally derived (19), from human RSV i.v. immunoglobulin (20, 21), or from palivizumab (22) are inversely correlated with RSV infection and the severity of RSV-related pneumonia. These include epidemiology studies demonstrating that RSV neutralizing titers of ≥7.64 to 8.6 log_2_ were associated with protection from bronchiolitis or pneumonia in young infants (19, 23). The high levels of neutralizing antibody titers attained by the administration of MEDI8897 support the concept that a single dose of MEDI8897 can provide protection for the duration of an RSV season.

In healthy adults, MEDI8897 had a favorable safety profile similar to placebo. MEDI8897 was well tolerated in study subjects, with similar types and frequencies of self-limited AEs reported in MEDI8897 and placebo recipients. Importantly, there were no SAEs or hypersensitivity events related to MEDI8897. In addition, the proportion of subjects with detectable ADA for MEDI8897 or placebo recipients was similar.

The findings of this study support further clinical development of MEDI8897 as a once-per-RSV-season prophylactic agent for the prevention of RSV-associated LRTI in infants.

## MATERIALS AND METHODS

### Study design.

This randomized, double-blind, dose-escalation, phase 1 study was conducted at a single site in the United States from April 2014 to June 2015 (registered at ClinicalTrials.gov under registration number NCT02114268). The study protocol, amendments, and informed-consent forms were reviewed and approved by an institutional review board (MidLands IRB, Overland Park, KS). This study was conducted in accordance with the Declaration of Helsinki and the International Conference on Harmonisation Guidance for Good Clinical Practice guidelines. Participants provided written informed consent before any study-related procedures were performed.

Following randomization, subjects received a single dose of placebo or MEDI8897, administered as an i.v. dose of 300 mg, 1,000 mg, or 3,000 mg or as an i.m. dose of 100 mg or 300 mg. The study enrolled 8 subjects for each cohort that received i.v. dosing and for the 100-mg i.m. cohort, while 104 subjects were enrolled in the 300-mg i.m. cohort. In each of these 5 cohorts, subjects were randomized 3:1 to receive MEDI8897 or placebo. Figure 1 provides subject numbers for each cohort. Since the study was conducted at a single site, manual randomization was used for assignment to each treatment group. An unblinded pharmacist was necessary to assign and dispense the investigational product. All other protocol-associated personnel, subjects, and clinical site staff were blinded to treatment assignments. The formulation for MEDI8897 was 100 mg/ml, and the appropriate volume of MEDI8897 was added to normal saline to achieve the desired volume for the i.v. dose cohorts. The total i.v. infusion volume was 50 ml for the 300-mg and 1,000-mg cohorts and 100 ml for the 3,000-mg cohort. The i.v. infusion time ranged from 20 min in the lowest-dose cohort to 200 min in the highest-dose cohort. For i.m. dosing, subjects in the 100-mg cohort received 1 i.m. injection of 1 ml, and subjects in the 300-mg cohort received a total of 3 ml administered as 3 separate i.m. injections. Following dosing, subjects were monitored for safety for approximately 360 days to correspond to the predicted 5 half-lives of the antibody.

### Participants.

Healthy, normotensive men or women who were 18 to 49 years of age with a body weight of 45 kg to 110 kg at screening were eligible for this study. Subjects were excluded from the study if they presented with an acute illness or a fever of ≥99.5°F at study entry, had previously received a monoclonal antibody, had received any drug therapy (except contraceptives) within 7 days prior to dosing or any vaccine within 14 days prior to dosing, had received any investigational therapy within 120 days prior to dosing through 360 days after dosing, had received immunoglobulin or blood products in the 6 months prior to study entry, or demonstrated clinically significant abnormal laboratory values at screening.

### Study assessments.

Pharmacokinetic parameters were evaluated using blood samples collected prior to dosing, at 8 h postdose, and at days 2, 4, 6, 8, 15, 22, 31, 61, 91, 121, 151, 181, 271, and 361. Samples were also collected at the end of infusion for subjects who received an i.v. dose. MEDI8897 serum concentrations were measured using a validated colorimetric enzyme-linked immunosorbent assay with a lower limit of quantitation (LLOQ) of 0.5 μg/ml. Antidrug antibody (ADA) was assessed using a validated electrochemiluminescent immunoassay, for which blood samples were collected prior to dosing and on days 15, 31, 91, 181, 271, and 361 after dose administration. Antidrug antibody titers of ≥1:50 were considered positive. Endogenous anti-RSV neutralizing antibody levels were determined using baseline serum samples collected predose in the MEDI8897 group and at predose and days 6, 31, 91, 181, and 361 in the placebo group. The sum of endogenous anti-RSV neutralizing antibodies and those afforded by MEDI8897 were measured at 8 h postdose and on days 6, 31, 91, 181, and 361 after dosing. An RSV A2-based microneutralization assay (24, 25) with an LLOQ of 1:10 or 3.32 log_2_ was used for this purpose. Neutralizing antibody titers were reported on a log_2_ scale. Safety was assessed by adverse events (AEs), serious adverse events (SAEs), adverse effects of special interest (AESIs), and new-onset chronic diseases (NOCDs) from the time of study drug administration through the end of the study. In this study, AESIs included hepatic function abnormalities, anaphylaxis, hypersensitivity and infusion reactions, immune complex disease, and thrombocytopenia. Investigators were instructed to report any laboratory and vital sign values that were deemed to be clinically significant as an AE.

### Statistical analyses.

As this study did not involve the statistical testing of a hypothesis, a formal sample size was not determined. The planned number of subjects was considered adequate to evaluate the objectives of this study, which were to determine PK, ADA, and safety and tolerability of MEDI8897 before initiating a study in infants. The placebo groups were pooled across all cohorts for analyses and presentation of results. Baseline was defined as day 1 (prior to dosing). Missing data were not imputed; in cases of missing data, only observed data were analyzed. Data were analyzed using SAS version 9.3 or higher (SAS Institute, Inc., Cary, NC) on a UNIX platform.

Pharmacokinetic parameters were estimated by noncompartmental analysis using Phoenix 64 WinNonlin 6.3 (Pharsight, Mountain View, CA). Values of the area under the concentration-time curve (AUC) were estimated using the linear trapezoidal rule with linear interpolation. Terminal half-life estimations using regression included at least 3 data points in the terminal linear phase of the semilog concentration-time profile. RSV antibody neutralization titers were summarized by mean (standard deviation [SD]) log_2_ 50% inhibitory concentrations and geometric mean fold rise for each treatment group (24). The proportion of subjects with a ≥4-fold rise in RSV neutralizing antibody titer from baseline and corresponding 95% Clopper-Pearson exact CI were summarized.
